# An Automated Method of Causal Inference of the Underlying Cause of Death of Citizens

**DOI:** 10.3390/life12081134

**Published:** 2022-07-28

**Authors:** Xu Yang, Hongsheng Ma, Keyan Gao, Hui Ge

**Affiliations:** 1 School of Computer Science and Technology, Beijing Institute of Technology, Beijing 100081, China; yangxu@tsinghua.edu.cn (X.Y.); 3220211176@bit.edu.cn (H.M.); 3220180795@bit.edu.cn (K.G.); 2 The Chinese Center for Disease Control and Prevention, Beijing 102206, China

**Keywords:** cause-of-death inference, confidence measurement, public heath, medical service

## Abstract

It is of great significance to correctly infer the underlying cause of death for citizens, especially under the current worldwide situation. The medical resources of all countries are overwhelmed under the impact of coronavirus disease 2019 (COVID-19) and countries need to allocate limited resources to the most suitable place. Traditionally, the cause-of-death inference relies on manual methods, which require a large resource cost and are not so efficient. To address the challenges, in this work, we present a mixed inference method named Sink-CF. The Sink-CF algorithm is based on confidence measurement and is used to automatically infer the underlying cause of death of citizens. The method proposed in this paper combines a mathematical statistics method and a collaborative filtering and analysis algorithm in machine learning. Thus, our method can not only effectively achieve a certain accuracy, but also does not rely on a large quantity of manually labeled data to continuously optimize the model, which can save computer computing power and time, and has the characteristics of being simple, easy and efficient. The experimental results show that our method generates a reasonable precision (93.82%) and recall (90.11%) and outperforms other state-of-the-art machine learning algorithms.

## 1. Introduction

Correctly inferring the underlying cause of death of citizens is always an important task. Especially since 2019, the outbreak of coronavirus disease 2019 (COVID-19) has been raging all over the world, which can be regarded as an extremely severe test for the health systems of all the countries. The ability to correctly infer and understand the underlying cause of death of citizens becomes critical and will help a country’s health system to rationalize and maximize the use of its very limited medical resources that are clearly under pressure in response to the COVID-19 epidemic.

Traditionally, the inference of the underlying cause of death of citizens is done through human work and relies highly on the experience of medical personnel, which has several disadvantages:

1. The accuracy of the inference results depends to a large extent on the medical staff’s experience;
2. As long as people participate in the work, it will inevitably be mixed with personal subjective judgment. The same medical staff may give different judgment as a result of the influence of different environments on the same case;
3. Without the help of an automatic approach, a large amount of manpower and time would be required. This is not an effective approach, especially under the current worldwide medical environment.

Some work has already been done towards the construction of an automatic method for the inference of the underlying cause of death of citizens [1,2,3]. As a result of the development of modern computer science technology, machine learning techniques began to be used in this process. However, the architecture of the cause-of-death chain, the complexity nature of the cause of death, and the required quantity of data in a cause-of-death dataset all restrict the effect and application of current techniques. In this paper, we introduce an automatic approach to help infer and identify the underlying cause of death of citizens. The method proposed in this paper combines a mathematical statistics method and a collaborative filtering and analysis algorithm in machine learning. Thus, our method can not only effectively achieve a certain accuracy, but also does not rely on a large quantity of manually labeled data to continuously optimize the model, which can save computer computing power and time, and has the characteristics of simple, easy and efficient.

The manuscript is arranged as follows: The literature is discussed in Section 2; the problem is defined and some basic concepts are introduced in Section 3; our method is presented in Section 4; the experiment results are shown and discussed in Section 5; the conclusion is given in Section 6.

## 2. Related Works

Many scholars have pointed out the disadvantages of inferring the underlying cause of death of citizens through only human work. Garne et al. analyzed the validity of the official cause of death regarding the registration of breast cancer patients in Malmö, Sweden, from 1964 to 1992. They revealed that the true cause of death differed from the officially reported cause of death in at least 4.6 percent of the deaths [4]. In 1998, Nalini pointed out that even the same cause-of-death chain could be inferred and determined with different results by different medical personnel when judging alone [5].

On one hand, this reinforces our belief that the medical staff’s judgment of the underlying cause of death depends largely on their experience. On another hand, this also hints that there are many factors that would influence the determination of the underlying cause of death beyond only the cause-of-death chain [6]. Thus, many researchers have put forward that the inference of the underlying cause of death should take into account other factors, such as the gender, physical health condition, age, habits and geographical characteristics of the case [7,8,9,10,11,12,13,14].

In 1991, Shaw-Hwa Lo proposed to construct incomplete an “cause-of-death chain” [15]. Boumezoued et al. used deterministic theory and random population dynamics models to construct a statistical model [16], to analyze cause-of-death mortality changes and its impacts on the whole population evolution. Bergeron-Boucher et al. provided a more detailed evaluation of the Lee–Carter (LC) model, by evaluating its accuracy, bias and robustness by age [17]. Their work also proved that gender factor must be taken into account when inferring the cause of death.

The development of modern computer science and data science has given us new power to build more efficient ways to do medical research. To comprehensively assess the predictive ability of several clinical variables for large-vessel occlusion (LVO) prediction, Tarkanyi et al. retrospectively analyzed prospectively collected multicenter stroke registry data. After a univariate analysis, they used the LASSO method for feature selection to select an optimal combination of variables, and various machine learning methods (random forest (RF), logistic regression (LR), elastic net method (ENM), and simple neural network (SNN)) were applied to optimize the performance of the model [18]. Jafari et al. used magnetic resonance imaging (MRI) and machine learning methods to diagnose the severity of brain tumors. They extracted the Gaussian and nonlinear scale features of MRI. The strongest features based on variance were selected and divided into 400 Gaussian and 400 nonlinear scale features, which were then mixed with each MRI. Finally, a classical machine learning classifier was used to check the performance of the proposed mixed-feature vectors [19]. To detect therapeutic biomarkers associated with respiratory-treatment response, Nikolaou et al. implemented several machine learning algorithms to predict the treatment response using identified biomarkers as well as age, sex, body mass index and lung function. These findings provide a valuable blueprint for why and how the use of biomarkers as diagnostic tools could prove helpful in guiding the therapeutic management of respiratory diseases [20].

Some research works about using machine learning methods to deal with cause-of-death inference have also been reported. For example, Danso et al. proposed to formalize the data from verbal autopsy documents and explore automatic text classification technology to obtain multiple causes of death automatically [21]. Deep learning technology was utilized by Falissard et al. to build an automatic inference model for the underlying cause of death, which obtained 75% accuracy [22]. The disadvantage of their work is the same as that in the study by Danso et al.

In 2021, Ge et al. presented an approach called the sink algorithm to achieve automatic inference of the underlying cause of death [2]. They treated the citizen as a boat on a river. Each cause of death was a stone that would be piled on that boat and finally cause the sink of that boat. They could achieve 87% accuracy.However, their methods had limitations. For example, their method’s effectiveness was reduced if no intuitive pathological causality was found in the cause-of-death chain.

## 3. Background and Concepts

### 3.1. Problem Definition

**Figure 1:**
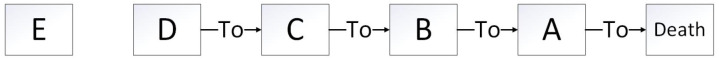
Illustration of cause-of-death chain.

A cause-of-death chain records the progression of a citizen’s disease or other things that finally lead to their death(Figure 1).

There are usually four items: A,B,C and D in a cause-of-death chain. Those items could be diseases, physical injuries or complications coded according to WHO (World Health Organization)’s ICD (International Classification of Diseases)-10 [23]. *A* is the direct cause of death. As we have discussed, sometimes, there are other things that are significantly implicated in the death of a citizen but not recorded in the chain, which are defined as *E* (Figure 1).

Thus, the issue of underlying cause-of-death inference is to decide the underlying cause of death from A,B,C,D, and E. There are two kinds of underlying cause of death: internal or external causes. If the underlying cause of death is from an external cause, then it might be the same as the direct cause of death [24,25].

In general, the underlying cause of death should be among A,B,C and *D*. However, it cannot be ruled out that in some cases, *E* is the underlying cause of death.

### 3.2. RDCP and CDCP

The Regional Death Cause Proportion (RDCP) is defined as:

(1) $$rdcp_{ij}=\frac{n_{ij}}{N_{j}},$$

where $rdcp_{ij}$ is the RDCP for cause *i* in region *j*, while $n_{ij}$, defined as the number of cases that cause *i*, is the underlying cause of death in region *j* and $N_{j}$ is defined as the number of death in region *j*.

The Conditional Death Cause Proportion (*CDCP*) is defined as:

(2) $$cdcp_{i}=P\left(S_{i}|T_{i}\right)=\frac{P(S_{i}T_{i})}{P(T_{i})},$$

where $cdcp_{i}$ is the conditional death cause proportion of cause *i*, while $S_{i}$ representing the event that causes *i* is the underlying cause of death, and $T_{i}$ representing the event that causes *i* is on the cause-of-death chain.

### 3.3. Sink Algorithm

The sink algorithm was developed under the motivation of mathematical statistics, where a citizen is treated as a boat on the river [2]. At first, he or she is a healthy citizen with different features (gender, region, and so on), similar to a boat in different style but pristine. As the citizen’s age grows, he or she suffers different diseases or other things, akin to different kinds of stone being piled in the boat. At last, the boat sinks representing the citizen dying.

As we would deduce, the sinking of the boat is the result of all the stones being piled on it. The underlying cause of death of a citizen is the stone that represents the most important cause of the boat’s sinking. There are also other factors that would influence a boat’s sinking, for example, the environment conditions, which might be a citizen’s medical history.

The sink algorithm defines the death process of a citizen as:

(3) R=A+B+C+D+E−ϵ(α+β+…),

where α,β and other parameters are used to describe the various status of a citizen and *R* represents that a citizen dies (or a boat sinks).

The underlying cause of death is the most severe element among A,B,C,D and *E*. The weight of a cause of death is defined as the CDCP of that cause. Furthermore, a more practical calculation method is given in [2] for CDCP as:

(4) $$CDCP_{i}=\log_{2}\frac{death\_count[i][age][gender]}{occur\_count[i][age][gender]}*10,$$

where death_count[i][age][gender] is defined as the number of samples where cause *i* is the underlying cause of death of that age group and gender group. occur_count[i][age][gender] is defined as the number of samples where cause *i* is a cause of death of that age group and gender group.

## 4. Sink-Cf Algorithm

### 4.1. Motivation

As a matter of fact, the four causes of death in the cause chain A,B,C and *D* often form an underlying causal chain. There is usually some implied pathological connection between the five causes A,B,C,D, and *E*. Over time, the causes that come first in the chain of cause of death are probably the cause of subsequent causes. The CDCP might be different for those causes. Therefore, it makes sense to select the largest disease prevention and control diseases as underlying cause of death [2].

However, the sink algorithm would become not so efficient under some special situations. For example, assume we have two cause-of-death chains:

1. Primary liver cancer (5 years) → aspiration pneumonia (gastric secretion) (2 days) → pulmonary infection (1 day) → infectious shock (6 h) → death
2. Pulmonary infection (2 years) → acute lower respiratory tract infection (6 days) → Ammetran allergy (1 h) → anaphylactic shock (20 min) → death

In both cases, the citizen died after shock. Furthermore, in both cases, a causality gap exists in the cause-of-death chain.

In case 1, the patient had a lung infection due to gastric secretion reflux into the respiratory tract, and eventually died of infectious shock. Therefore, the underlying cause of death should be aspiration pneumonia. However, the sink algorithm would conclude that the primary liver cancer is the underlying cause of death because the primary liver cancer has a higher CDCP value.

In case 2, because the CDCP of the acute lower respiratory tract infection is higher than the Ammetran allergy, the sink algorithm would decide that acute lower respiratory tract infection was the underlying cause of death. In fact, although in this case the patient developed an acute lower respiratory tract infection due to pulmonary infection, he/she died from anaphylactic shock caused by misusing Ammetran. Therefore, the underlying cause of death should be Ammetran allergy.

There are other situations that would also cause the sink algorithm to be not so efficient. In order to improve the sink algorithm, we propose the Sink-CF (sink collaborative filtering) algorithm.

The idea is to use the sink algorithm and collaborative filtering to complement each other to construct a more effective method of automatic underlying cause-of-death determination. On the one hand, the sink algorithm makes up for the deficiency of the collaborative filtering method with few characteristics and wide classification dimensions; on the other hand, the collaborative filtering method solves the deficiency of the sink algorithm in the face of special circumstances.

### 4.2. Confidence Measurement Based Sink-CF Algorithm

**Figure 2:**
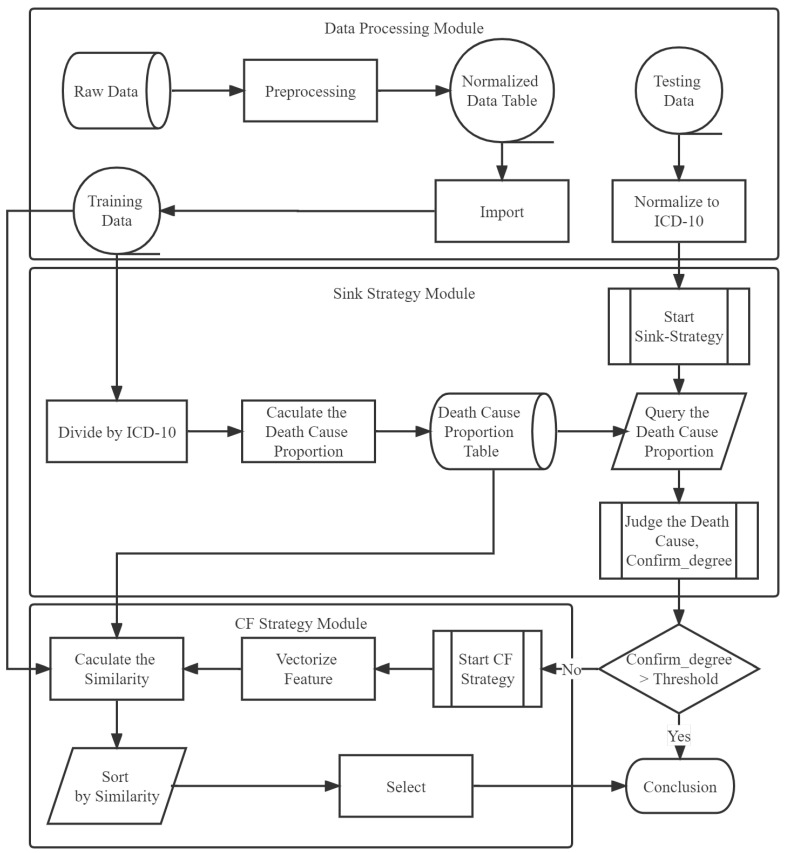
The confidence measurement based on the Sink-CF algorithm.

Sink-CF is a mixed inference model. This work constructs this mixed inference model based on confidence measurement, as shown in Figure 2.

The sink algorithm declares the cause of death with highest CDCP as the underlying cause of death. This method’s efficiency would be challenged when more than 1 causes of death have a higher CDCP, or we could say that the confidence of the sink algorithm decreases when the CDCP values of all causes of death converge.

As for collaborative filtering, since it needs to be inferred from historical cases, the number and similarity of similar historical cases will affect the confidence of the algorithm. The more historical the cases, the more similar to the characteristics of the cases to be inferred, the more persuasive the inference results are.

#### 4.2.1. Sink Strategy Module

For the cause-of-death chain represented as Equation (3), we calculate the weight of each cause of death according to Equation (4). The citizens are divided into three groups by age: [0,18], [18,55] and [55,∞]. Gender groups are male and female. The cause of death with the highest CDCP value would be declared as the result of the sink algorithm.

The confidence measurement of the sink algorithm is defined as $C_{Sink}$ and is calculated as:

(5) $$C_{Sink}=\sin\left(\frac{\pi }{2}*n\right),$$

where *n* is the number of causes in the cause-of-death chain that have weighted CDCP values larger than a predefined threshold value. In this work, the threshold value was defined as the average value of the weighted CDCP value of all the causes in the cause-of-death chain. The weighted CDCP value is calculated with respect to the number of occurrences of that cause in the dataset. If $C_{Sink}=1$, then the confidence of the sink algorithm is very high, and we can use the result of the sink algorithm as the final result of Sink-CF. If the result of $C_{Sink}$ is 0 or −1, then we need the verification from the CF Strategy module.

#### 4.2.2. CF Strategy Module

The Jaccard similarity measurement is used to measure the similarity between different cause-of-death chains. Because the available features are very limited in the cause-of-death chain analysis, it means that the calculation of the similarity in the collaborative filtering analysis produces a huge difference between “complete equality” and “very similar”, which should belong to the approximate relationship. Thus, the standard Jaccard similarity measurement method is not suitable.

We believe that by combining the CDCP values of different causes, as the weight of the vector projection in various dimensions when calculating the similarity process, it can effectively increase the similarity difference between different samples and improve the efficiency of the similarity calculation. The idea behind this is the same as in the sink algorithm: a higher CDCP means that the cause is more harmful to a citizen and is more likely to be the underlying cause of death of the citizen. When the same type of cause with a relatively high CDCP occurs in two cases, it can be considered that the cause has an enhanced effect on the similarity of the two cases; conversely, if the CDCP value of that cause is relatively low, even if it appears in the corresponding location in two cases, it may not have a significant effect on the similarity of the two cases.

Thus, in this work, the similarity between cause-of-death chain $C_{a}$ and $C_{b}$ was calculated as:

(6) $$sim\left(C_{a},C_{b}\right)=J\left(I_{a},I_{b}\right)=\frac{\left|(I_{a}\cap I_{b})\cdot R\right|}{\left|(I_{a}\cup I_{b})\cdot R\right|},$$

where $I_{a}$ and $I_{b}$ represent the set comprised of the causes in chain *a* and chain *b*, respectively, while the weight matrix *R* was defined as:

(7) $$R=\left(\begin{array}{ccc} r_{a_{1}}r_{b_{1}} & \cdots & \cdots \\ \vdots & \ddots & \vdots \\ \cdots & \cdots & r_{a_{n}}r_{b_{n}} \end{array}\right),$$

where $r_{a_{i}}$ is the CDCP value of cause $a_{i}$ in chain *a*, and $r_{b_{i}}$ is the CDCP value of cause $b_{i}$ in chain *b*.

**Figure 3:**
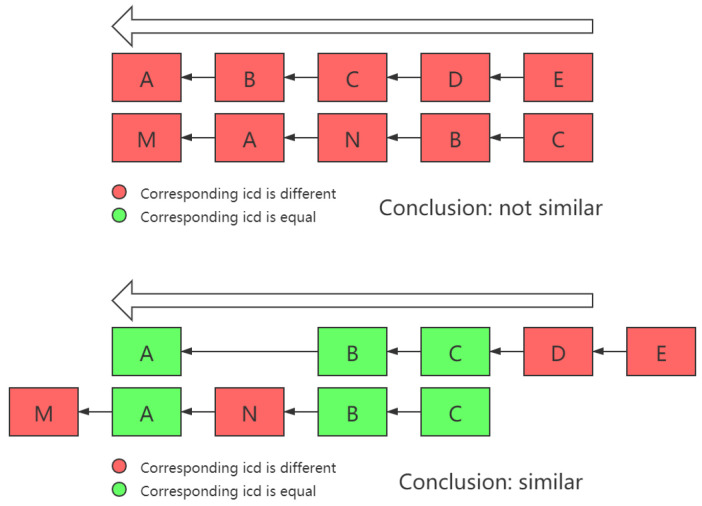
Location or consequences are different.

Another thing that needs to be noted is that sometimes, there are similar patterns in two chains, but due to certain reasons, they appear in different locations or consequences in two chains, as shown in Figure 3.

In order to deal with this issue, in the process of calculating the similarity between different cases, the problem of whether the projection of different dimensions of two vectors falls into the same axis must be solved. Therefore, in this work, the intersection of $I_{a}$ and $I_{b}$ was no longer directly used as the vector intersection operation, but a dimension transposition was carried out first, in order to maximize the total number of dimensions projected on the same dimension on the two cause-of-death chains.

## 5. Experiments and Results

### 5.1. Experiment Framework

The experiments were carried on a hardware platform comprising Intel i7-9700 processors, 16 GB DDR4 memory, 512 GB hard disk. The database was MySQL 8.0. Furthermore, the version of Python was 3.5.

The dataset included nearly 50,000 records from a northern Chinese city.

### 5.2. Results

In our experiment, we input the whole cause-of-death chain and some other factors in the record, as shown in Figure 1, and asked the method to infer the underlying cause of death. We used the cross-validation method to verify the effect of our method. The whole dataset was divided into 17 parts, 16 of which formed the training set and 1 was the test set. Each turn, 16 subsets were used to train the algorithm while 1 subset was used as the test set. After 16 turns, the average results were used as the final result.

First, we calculated the weighted CDCP values for all causes. According to our analysis, around 64.35% of the samples in the dataset could generate a result directly by the sink strategy module. The CF strategy module needed to be invoked for the remainder.

After each round of cross-validation, we calculated the number of samples correctly inferred, and obtained the average value after 16 rounds of cross-validation. We divided the average number of samples correctly inferred by the total number of samples as the accuracy of the final inference. Furthermore, the final result of the accuracy was 91.81%.

**Figure 4:**
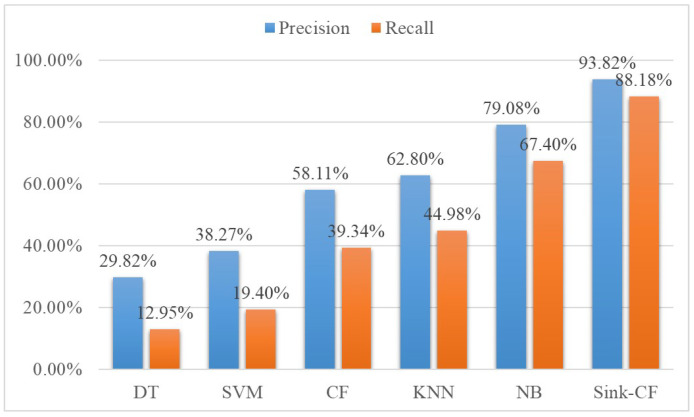
Comparison with state-of-the-art methods.

To prove the results of our Sink-CF algorithm based on confidence measures, several state-of-the-art classification methods were chosen to compare: DT (decision tree), SVM (support vector machine), CF (collaborative filter), etc. The Sink-CF algorithm could obtain the highest precision (93.82%) and recall (90.11%). This validated that our method could achieve optimal performance. The comparison result is shown in Figure 4.

**Figure 5:**
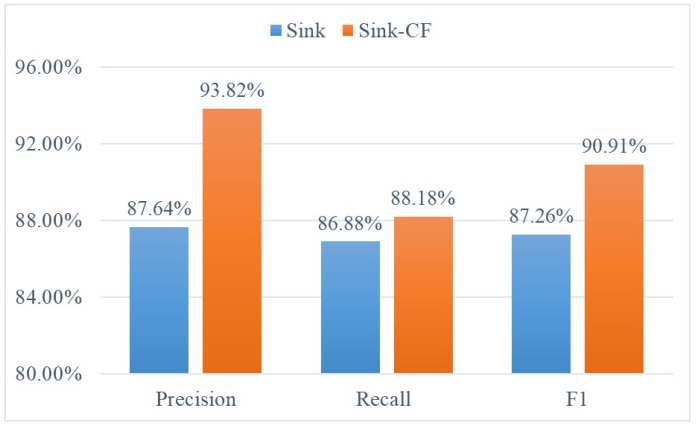
Comparison with the sink algorithm.

The comparison result with Sink algorithm is shown in Figure 5.

**Figure 6:**
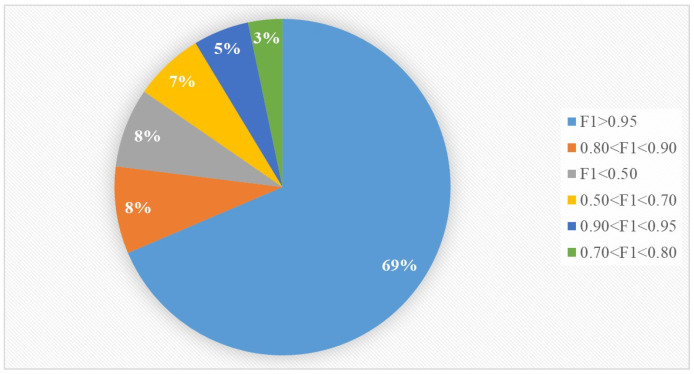
F1-score for different ICD-10 causes.

We used the F1-score as the final metric to assess the generalizability of our approach for different causes. The result obtained is shown in Figure 6.

The F1-score was larger than 0.95 for 69% of causes. In only 14.37% of causes was the F1-score less than 0.7. Thus, the high generalizability could be proved.

## 6. Conclusions

Inferring the underlying cause of death has long been a challenge for primary medical workers. To correctly grasp the cause of death of citizens is not only the basic requirements of medical and health work, but also to provide a data basis for medical data research, residents’ health status and public health level. In this work, we proposed a confidence-measurement-based Sink-CF algorithm, by introducing a collaborative filtering method to improve the sink algorithm, thus establishing a practical and generalized automatic inference method for the underlying cause of death of citizens.

We used several STOA machine learning methods to compare with our Sink-CF algorithm the inference of the underlying cause of death of citizens. The DT method used age, gender and cause-of-death composition as input, using one of them as root node, and the leaf node was the underlying cause of death. However, due to the small number of items in individual cases, the constructed tree was too shallow. It did not have sufficient power to deal with the inference of the underlying cause of death. As for the SVM, the motivation was to maximally distinguish the citizens of different underlying causes of death to define the inference of the underlying cause of death as a solution of a convex quadratic programming issue. However, the number of possible underlying causes of death was too huge, thus it was impossible to generate reasonable results with the limited number of input samples. The CF method followed the idea of “finding similarities and connections”. However, in the case of limited input data samples, relying only on CF to infer the underlying cause of death was not wise. The essence of the KNN was also to find similarities in the datasets. However, on the one hand, its inference effect greatly depended on the amount of similar data. In the face of some rare cases, a poor inference effect occurred due to the small quantity of similar data. On the other hand, the measurement of the calculated distance was not easy to set up, because the effect of different characteristics on the cause of death varied greatly, which affected its application. Using the NB method to implement inference of the underlying cause of death had high requirements on the dataset and had the limitation caused by the inconsistency between the principle of NB with the requirement of the dataset, which also restricted the effect. The sink method could deal with those issues, generating reasonable results. However, under certain cases, the method’s effectiveness was limited.

With the help of a collaborative filter, our Sink-CF method could largely enhance the inference accuracy compared with other state-of-the-art methods. In future work, we will start with the cause-of-death chain sequence relationship, from a continuous, holistic perspective to think about the factors that cause death, mining the relationship between various causes in big data, introducing artificial intelligence method to further improve the efficiency of the proposed algorithm.

## Data Availability

Not applicable.
